# Effects of Heparin and Bivalirudin on Thrombin-Induced Platelet Activation: Differential Modulation of PAR Signaling Drives Divergent Prothrombotic Responses

**DOI:** 10.3389/fcvm.2021.717835

**Published:** 2021-09-29

**Authors:** Mikael Lund, Ankit S. Macwan, Kjersti Tunströmer, Tomas L. Lindahl, Niklas Boknäs

**Affiliations:** ^1^Department of Biomedical and Clinical Sciences, Linköping University, Linköping, Sweden; ^2^Department of Clinical Chemistry and Department of Biomedical and Clinical Sciences, Linköping University, Linköping, Sweden; ^3^Department of Hematology and Department of Biomedical and Clinical Sciences, Linköping University, Linköping, Sweden

**Keywords:** heparin, bivalirudin, thrombin, PAR1, PAR4

## Abstract

Heparin and bivalirudin are widely used as anticoagulants in the setting of acute thrombosis. In this study, we investigated how these drugs affect the ability of thrombin to generate a prothrombotic platelet response via activation of the protease-activated receptors (PARs) 1 and 4. We examined the effects of heparin/antithrombin and bivalirudin on PAR1- and PAR4-mediated intracellular calcium mobilization, aggregation, α-granule release, and procoagulant membrane exposure in platelets exposed to thrombin concentrations likely to be encountered in the thrombus microenvironment during thrombosis. At physiological antithrombin levels, heparin treatment resulted in complete and sustained inhibition of thrombin-induced PAR4-mediated platelet activation, but transient PAR1 signaling was sufficient to elicit significant α-granule release and platelet aggregation. In contrast, bivalirudin treatment resulted in rapid and profound inhibition of signaling from both PAR receptors, followed by a delayed phase of PAR4-mediated platelet activation, resulting in a robust prothrombotic response. Combination treatment with bivalirudin and subtherapeutic concentrations of heparin completely inhibited the residual platelet activation observed with single drug treatment at all time-points. Our results show that heparin and bivalirudin have different and complementary inhibitory effects on the activation of PAR1 and PAR4 by thrombin.

## Introduction

With the growing use of life-saving invasive treatments such as ventricular assist devices, extracorporeal membrane oxygenation, open heart surgery and endovascular interventions, patients are increasingly subjected to situations exposing them to a very high risk of both bleeding and thrombosis. For such treatments to be successful, it is imperative to exert a tight control of the hemostatic system. Empirically, such control has been achieved by treatment with a thrombin inhibitor such as heparin or bivalirudin, often in combination with one or several anti-platelet agents. One prototypic example is percutaneous coronary intervention (PCI). While timely PCI is critical for achieving reperfusion of the ischemic myocardium in the setting of acute coronary syndromes, the procedure itself puts patients at high risk for thrombotic events. Thus, a potent antithrombotic combination therapy including at least two antiplatelet agents and one anticoagulant is administered peri-procedurally as prophylaxis (1). Several large clinical studies have been performed to assess whether heparin or bivalirudin is associated with the best outcome in this context. Although results are somewhat conflicting (2, 3) the use of bivalirudin has often been associated with lower rates of bleeding at the cost of a higher incidence of stent thrombosis (4–6).

Heparin refers to unfractionated preparations of glycosaminoglycan polymers of different sizes, which avidly bind to antithrombin and exert their antithrombin activity by simultaneous interactions with thrombin's exosite II, thereby serving as templates for the formation of a ternary complex which irreversibly inactivates the protease (7). Bivalirudin on the other hand, is a rationally designed peptide comprising the sequence D-Phe-Pro-Arg-Pro that binds directly to the active site of thrombin, but also a hirudin-like sequence which binds to exosite I, the fibrinogen recognition site. In its native configuration, bivalirudin thus acts as a non-competitive bivalent thrombin inhibitor. However, bivalirudin's inhibitory effects on thrombin are changed over time, as thrombin gradually cleaves the Arg_3_-Pro_4_ bond, turning the molecule into a competitive thrombin inhibitor that retains binding to exosite I but leaves thrombin's active site free to interact with substrates (8).

Apart from catalyzing fibrin polymerization, thrombin also exerts its prothrombotic effects by potently activating platelets via the protease-activated receptors (PARs) 1 and 4. Despite being structurally very similar, a growing body of evidence point toward different roles of PAR1 and PAR4 in platelet physiology. Due to the presence of a hirudin-like motif conferring high-affinity interactions with exosite I, PAR1 is activated at low thrombin concentrations, resulting in a rapid but transient rise in intracellular calcium concentrations with ensuing platelet aggregation and granule secretion (9). In contrast, activation of PAR4 involves exosite II (10), occurs at higher thrombin concentrations, and is associated with platelet activities that require more sustained calcium mobilization, such as procoagulant membrane exposure (11, 12) and platelet spreading (13). As exosites I and II have been shown to be crucial for determining thrombin substrate recognition (14, 15), the differential engagement of these thrombin domains by heparin and bivalirudin could result in alterations in substrate specificity that might have consequences for the pharmacodynamic effects of these drugs. This concept is supported by the previous finding that a synthetic low molecular weight heparin analog without anticoagulant activity selectively inhibited PAR4-mediated platelet aggregation at low thrombin concentrations (16). The purpose of this study was to test the hypothesis that the different mechanisms employed by unfractionated heparin/antithrombin and bivalirudin to inhibit thrombin result in differential inhibition of thrombin-induced platelet activation responses via PAR1 and PAR4.

## Materials and Methods

### Materials

Protease-activated receptor activating peptides SFLLRN (PAR1-AP) and AYPGKF (PAR4-AP) were from JPT Peptide Technologies GmbH (Berlin, Germany). Human α-thrombin, apyrase, PGI_2_, and Corning® 96-well high content imaging glass bottom microplates were from Sigma-Aldrich (St. Louis, MO, US). Vorapaxar, a selective PAR1-antagonist, was from AdooQ Bioscience (Irvine, CA, US), and BMS-986120, a selective PAR4-antagonist, was from MedKoo Biosciences Inc (Morrisville, NC, US). Unfractionated heparin (UFH) was from LEO Pharma (Ballerup, Denmark), and antithrombin was from Baxalta Innovations (Wien, Austria). Bivalirudin was from The Medicines Company UK Ltd (Abingdon, Oxfordshire, UK). Dabigatran was purchased from Selleckchem (Munich, Germany). The calcium binding dye Fluo-4 AM, Annexin V, Alexa Fluor™ 647 conjugate, and Alexa Fluor™ 555 antibody labeling kit were from Thermo Fisher Scientific (Waltham, MA, US). Monoclonal antibodies for flow cytometry [anti-P-selectin-allophycocyanin (anti-CD62P-APC, clone AK4), PAC-1-fluorescein isothiocyanate (FITC), and an APC isotype control antibody (mouse IgG1κ), were from BD Biosciences (San Jose, CA, US). Anti-CD42b-R-phycoerythrin (R-PE) was from DAKO (Glostrup, Denmark). For microscopy, anti-human CD41 antibody, clone PM6/248 was from Bio-Rad Laboratories AB (Solna, Sweden), and calcium binding dye, Cal-520® AM, was from AAT Bioquest (Sunnyvale, US). HEPES (N-2-hydroxyethylpiperazine-N′-2-ethanesulfonic acid) buffer (137 mmol/L NaCl, 2.7 mmol/L KCl, 1 mmol/L MgCl_2_, 5.6 mmol/L glucose, 1 g/L bovine serum albumin, and 20 mmol/L HEPES, pH 7.40) was used either with or without supplementation of 1.5 mmol/L CaCl_2_ (denoted HEPES-Ca^2+^). HEPES buffered saline (HBS; 154 mmol/L NaCl and 20 mmol/L HEPES, pH 7.4) and Krebs Ringer Glucose (KRG; 120 mmol/L NaCl, 4.9 mmol/L KCl, 1.2 mmol/L MgSO_4_, 1.7 mmol/L KH_2_PO_4_, 8.3 mmol/L Na_2_HPO_4_, 10 mmol/L glucose, pH 7.3) were also used in sample preparations. Phosphate buffered saline (PBS) with 0.2% formaldehyde was used to fix platelets. All reagents were stored and used according to the manufacturers' recommendations.

### Blood Collection and Sample Preparation

Venous blood from healthy adult volunteers was collected in 9 mL acid-citrate-dextrose tubes from Greiner Bio-One GmbH (Kremünster, Austria). The gender distribution among study participants was ~1:1 and the age range 18–65 years. Study participants with chronic medical conditions or any consumption of drugs known to interfere with platelet function (e.g., Aspirin®) for a period of 10 days prior to study recruitment were excluded from further participation. Informed consent was acquired in accordance with the Declaration of Helsinki, and the procedure used anonymised samples as required in the approval decision by the local ethics committee in Linköping, Sweden (ethical permission numbers 2012/382-31 and 2016/314-32).

Blood samples were left to rest at room temperature for 30 min before platelet rich plasma (PRP) was collected by centrifugation for 15 min at 150 g. Apyrase (1 U/mL) was added, and PRP was centrifuged at 480 g for 20 min. The platelet pellet was re-suspended in KRG buffer supplemented with PGI_2_ (100 nmol/L), after which the platelet count was adjusted to 250 × 10^9^ cells/L. Isolated platelets were then incubated for 30 min at RT. For subsequent experiments, platelet suspensions were incubated 10 min with physiological levels of antithrombin (1 U/mL) and combinations of vorapaxar (VPX, 5 μmol/L), BMS-986120 (BMS, 100 nmol/L), heparin, or bivalirudin. Bivalirudin concentrations of 1 μg/mL were selected to mimic plasma concentrations ~1–2 h post-PCI in patients treated with the 0.75/1.75 bivalirudin dosage regimen (17). The heparin concentrations used (0.2 and 1.0 U/mL) correspond to the low and high extremes of anti-FXa levels detected in patients during therapeutic heparin dosing (18). To mimic local conditions in the thrombus micro-environment during acute thrombosis (19), the α-thrombin concentration was set to 4 U/mL (34 nmol/L) in all experiments.

### Measurement of Intracellular Calcium

Intracellular calcium mobilization was assessed by a spectrofluorometric method using an Enspire™ fluorescence plate reader (PerkinElmer, Waltham, US). Isolated platelets were diluted to 50 × 10^9^ cells/L in HBS-buffer and incubated with the calcium indicator Fluo-4 AM (5 μmol/L) for 20 min at RT. Inhibitors were added in specified combinations and experiments were performed at 37°C in a Nunc™ 384-well clear polystyrene microtiter plate (Thermo Fisher Scientific, Waltham, MA, US). After registering an initial baseline, platelets were activated by automatic dispensation of α-thrombin, PAR1-AP or PAR4-AP at indicated concentrations. Fluorescence was measured in 5 s intervals for 120 or 480 cycles. Excitation was set to 495 nm and emission was recorded at 535 nm.

### Light Transmission Aggregometry

Aliquots (0.3 mL) of suspensions with washed platelets (250 × 10^9^ cells/L) were incubated with antithrombin and selected inhibitors for 10 min at RT. After incubation at 37°C for 2 min, platelet aggregation was induced by manual addition of 4 U/mL α-thrombin. Changes in light transmission were recorded with a Chrono-log Corporation model 490 X-aggregometer (Havertown, PA, US).

### Flow Cytometry

Flow cytometry was performed on a Gallios™ flow cytometer (Beckman Coulter Inc., Fullerton, CA, US) as described earlier (20, 21) with minor modifications. Washed platelets were incubated with inhibitors as described above. Anti-CD42b-RPE (2.5 μg/mL), PAC-1-FITC (0.56 μg/mL), and anti-CD62P-APC (1.25 μg/mL) were used for staining. An APC-labeled IgG1 isotype control antibody was included as a negative control for CD62P (1.25 μg/mL). Experiments were performed in HEPES-Ca^2+^ or HEPES-EDTA buffer (isotype only), and stopped after 20, 25, 30, 35, 40, and 45 min through dilution 1:20 with PBS supplemented with 1 μg/mL dabigatran and 0.2 % formaldehyde. Forward scatter and anti-CD42b fluorescence were used to gate the washed platelets. Platelet sample containing HEPES-EDTA buffer and Isotype-APC antibody was used to set up the background fluorescence level, which was set to ≤ 2%. Data was acquired until reaching 10,000 platelet events or 60 s. Median fluorescence intensity (MFI) was analyzed using Kaluza Analysis v.1.3 (Beckman Coulter).

### Microscopy

Platelets were imaged on a Nikon Eclipse Ti confocal microscope with a CrEST spinning disk system using the X 60 NA 0.8 lens. Briefly, washed platelets (200 × 10^9^/L) were stained with the calcium dye Cal-520 AM at 37°C for 45 min, washed (1,500 g for 90 s), resuspended in HEPES-Ca^2+^, and stained with a platelet identification marker (anti-CD41 antibody (1:100) conjugated with Alexa flour 555) for 10 min. Fibrinogen (1 mg/mL) was coated in a well of a high content imaging glass bottom microplate, and the non-coated surface was blocked by 1% bovine serum albumin. Suspensions of stained platelets were then incubated in the fibrinogen coated well for 10 min with antithrombin (1 U/mL) and combinations of heparin (1 U/mL) or bivalirudin (1 μg/mL) as indicated. Excess platelet suspension was washed off and wells were supplemented with buffer containing annexin V-AF647 conjugate (1:100). Thrombin (4 U/mL) was added at the start of most experiments, and time-lapse images were acquired every 15 s for 45 min. Offline data analysis was performed with ImageJ (22).

### Data Analysis and Statistics

Data for calcium measurements are presented after normalization and correction for baseline drift with QtiPlot v.0.9.9 (https://www.qtiplot.com). Normalization was performed by setting the peak response with 4 U/mL thrombin to 100%. Data obtained from aggregometry and flow cytometry was not normalized. Analysis of variance (one-way ANOVA) followed by Dunnett's multiple comparisons test was performed using GraphPad Prism version 8.0.1 for macOS (GraphPad Software, San Diego, CA, US, www.graphpad.com). *P* < 0.05 was considered statistically significant.

## Results

### Heparin Preferentially Inhibits PAR4 Activation by Thrombin

**Figure 1:**
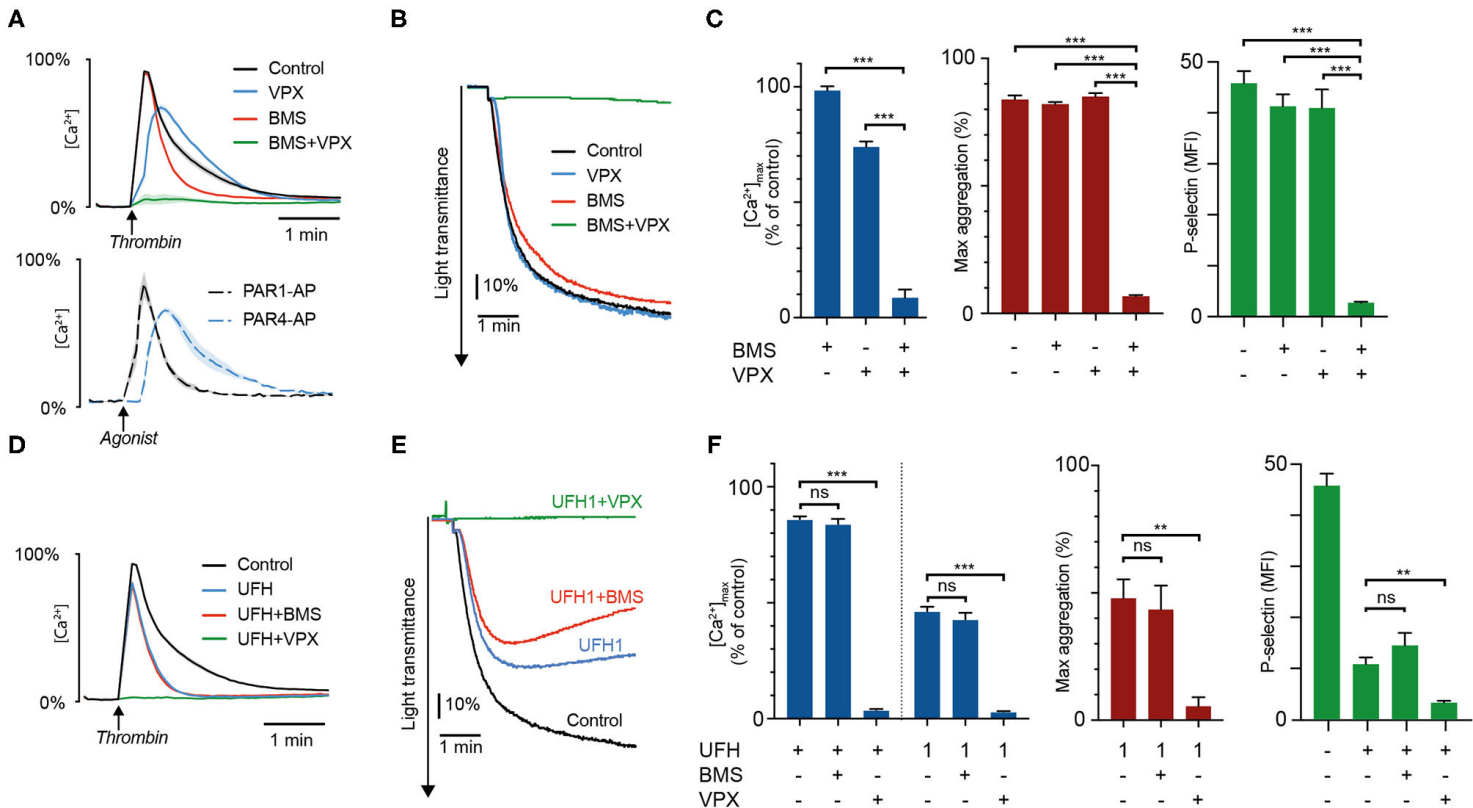
Heparin preferentially inhibits thrombin-induced platelet activation via PAR4. Intracellular calcium mobilization, platelet aggregation, and P-selectin exposure was measured in suspensions of washed platelets supplemented with 1 U/mL antithrombin after addition of the PAR1-inhibitor vorapaxar (5 μmol/L), the PAR4-inhibitor BMS-986120 (100 nmol/L), or heparin (0.2 U/mL, unless specified as 1 U/mL). Platelets were activated with 4 U/mL α-thrombin. **(A–C)** show the inhibitory effects of PAR-inhibitors alone and in combination. Calcium profiles generated with PAR1-AP (15 μM) and PAR4-AP (150 μM) are presented for comparison. **(D–F)** show the effects of heparin ± PAR inhibitors. Results represent the mean ± SEM (Calcium measurements: Control, VPX, *n* = 12; BMS, BMS+VPX, *n* = 8; UFH, *n* = 11; UFH+BMS, *n* = 7; UFH+VPX, *n* = 6; PAR1- and PAR4-AP, *n* = 3. LTA: In **(C)** Control, *n* = 10; BMS, *n* = 6; VPX, *n* = 9; BMS+VPX, *n* = 5. In **(F)** UFH, *n* = 8; UFH+BMS, UFH+VPX, *n* = 4. Flow cytometry: Control, *n* = 12; BMS, VPX, UFH, *n* = 10; BMS+VPX, *n* = 9; UFH+BMS, UFH+VPX, *n* = 4). **(B,E)** show representative data. **P < 0.01; ***P < 0.001; not significant (ns). VPX, vorapaxar; BMS, BMS-986120; UFH, unfractionated heparin.

Vorapaxar (VPX) and BMS-986120 (BMS) are highly specific and potent inhibitors of thrombin-induced proteolytic activation of PAR1 and PAR4, exerting their inhibitory activity by competitive binding to the targeted receptor. Since these drugs do not affect the proteolytic activity of thrombin with regards to other substrates (23–25), we hypothesized that pre-treatment of platelets with VPX and BMS would allow us to determine the effects of thrombin inhibitors on the activation of PAR4 and PAR1, respectively. To validate this concept, we compared the intracellular calcium transients triggered by thrombin in the presence of VPX+/– BMS with results obtained using the specific PAR activating peptides PAR1-AP and PAR4-AP. As shown in Figure 1A, pre-treatment with VPX or BMS resulted in calcium transients closely resembling those provoked by saturating concentrations of PAR4-AP and PAR1-AP, respectively. Additionally, whereas treatment with either PAR inhibitor only slightly reduced calcium mobilization induced by 4 U/mL thrombin, combination treatment resulted in profound inhibition of calcium mobilization, platelet aggregation and P-selectin exposure (Figures 1A–C). Together, these findings verified the inhibitory specificity and potency of VPX and BMS in our assays and showed that these drugs could be used to isolate the individual contributions of PAR1 and PAR4 to thrombin-induced platelet activation.

Platelet exposure to thrombin after pre-treatment with heparin gave rise to calcium “spikes” with slightly reduced amplitudes but substantially shortened signal duration (Figure 1D). These calcium profiles closely resembled those produced by either PAR1-AP, or thrombin in presence of the PAR4-inhibitor BMS, suggesting that the residual platelet activation observed in the presence of heparin was mainly derived from PAR1. We confirmed the validity of this observation through experiments with heparin and combinations of VPX and BMS. No additional effect could be observed on calcium mobilization, platelet aggregation, and P-selectin exposure when BMS was added in combination with heparin, but pronounced additive inhibition was observed with a combination of vorapaxar and heparin at therapeutic and subtherapeutic concentrations (1 and 0.2 U/mL, Figures 1E,F and Supplementary Figure 1A).

### Thrombin Inhibition With Bivalirudin Results in a Delayed Prothrombotic Response Phase Mediated by PAR4

**Figure 2:**
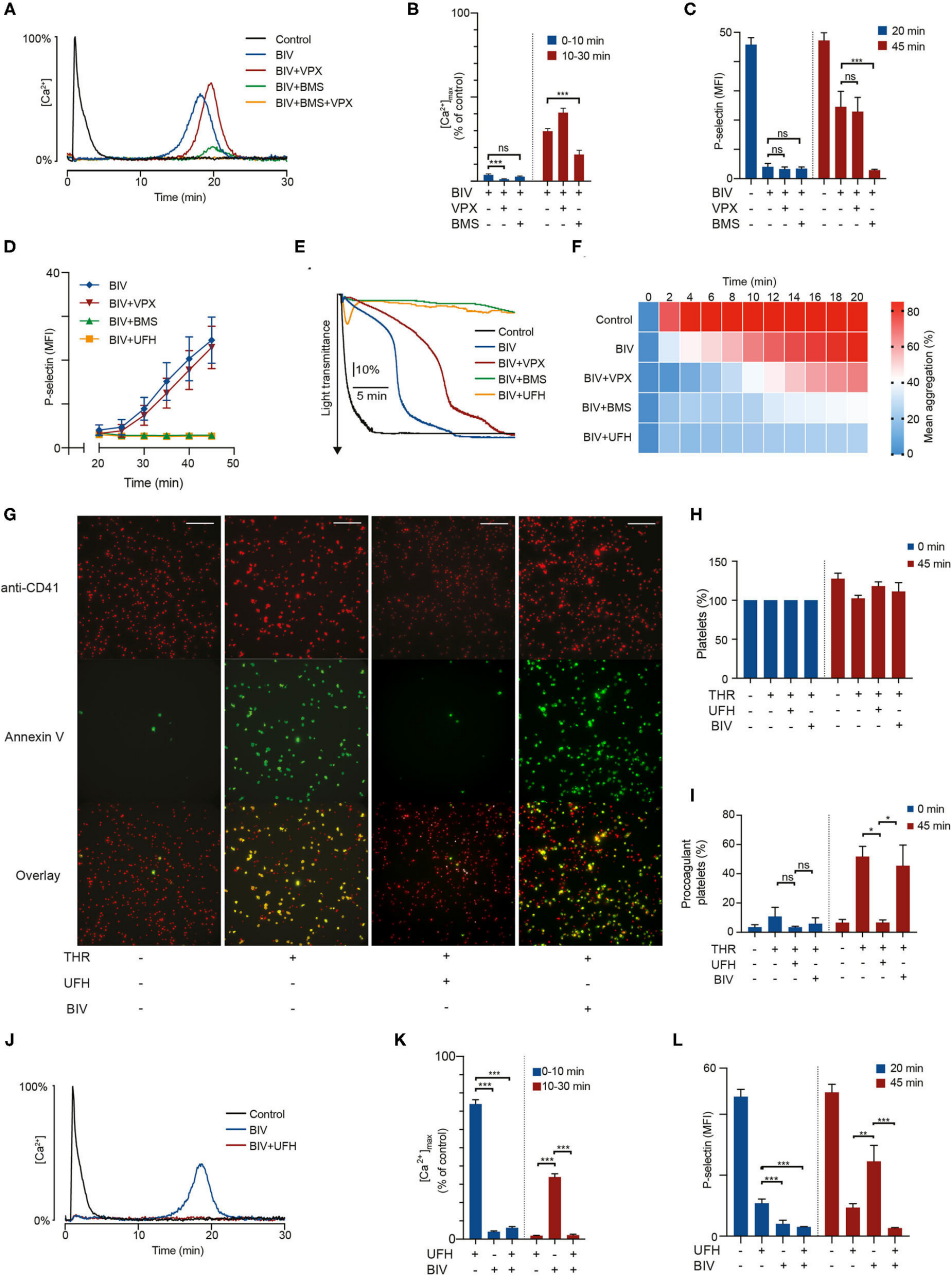
PAR4 activation triggers a delayed prothrombotic response in bivalirudin-treated platelets exposed to thrombin which can be suppressed by sub-therapeutic doses of heparin. Suspensions of washed platelets supplemented with 1 U/mL antithrombin were incubated for 10 min with vorapaxar (5 μmol/L), BMS-986120 (100 nmol/L), heparin (0.2 U/mL), or bivalirudin (1 μg/mL) as indicated, followed by exposure to 4 U/mL α-thrombin. In **(A,B,J,K)**, intracellular calcium mobilization was monitored continuously for 30 min using the calcium probe Fluo-4. Data is presented as a fraction of the maximum fluorescence intensity registered after α-thrombin stimulation (4 U/mL). In **(C,D,L)** aliquots of platelets were incubated after thrombin treatment as indicated and then analyzed with flow cytometry for P-selectin exposure to assess α-granule release. Data is presented as mean fluorescence intensity (MFI). In **(E,F)** platelet aggregation was monitored continuously for 20 min using LTA. In **(G–I)** platelets stained with a fluorescent platelet marker (red) were allowed to spread on fibrinogen-coated wells for 15 min before treatment as indicated, and then monitored for Annexin V-binding (green) as a marker for procoagulant membrane exposure using confocal microscopy. Pictures are taken after 45 min. Scalebar represents 50 μm. Results show the mean ± SEM (Calcium measurements: Control, BIV, *n* = 21; BIV+VPX, *n* = 18; BIV+BMS, *n* = 13; UFH, BIV+UFH, *n* = 11. LTA: Control, BIV+VPX, BIV+UFH, *n* = 8; BIV, *n* = 13; BIV+BMS, *n* = 9. Flow cytometry: Control, BIV, BIV+VPX, BIV+BMS, BIV+UFH, *n* = 12; UFH, *n* = 10. Microscopy: THR, BIV, *n* = 4. UFH, *n* = 3). (**A,E,G,J**) show representative data. *P < 0.05; **P < 0.01; ***P < 0.001; not significant (ns). THR, thrombin; VPX, vorapaxar; BMS, BMS-986120; UFH, unfractionated heparin; BIV, bivalirudin.

Treatment with 1 μg/mL bivalirudin produced rapid and pronounced inhibition of thrombin-induced calcium mobilization in the time range 0–10 min (Figures 2A,B and Supplementary Figure 1B). However, we observed a delayed activation phase occurring after 10–45 min, with large variations in time of onset depending on assay and individual donor. This peculiar pattern of intracellular calcium signaling translated into markedly delayed α-granule exocytosis, with essentially no P-selectin exposure after 20 min of thrombin stimulation followed by a gradual increase at 20–45 min to around 50% of the expression levels observed without bivalirudin (Figures 2C,D). Bivalirudin treatment was also associated with a significant delay in platelet GPIIb/IIIa-activation and aggregation responses to thrombin (Figures 2E,F and Supplementary Figures 2A,B). Yet, the inhibitory potency of bivalirudin seemed to wane faster in aggregometry, perhaps due to reinforcement of platelet activation related to outside-in signaling by the fibrinogen receptor (GpIIb/IIIa) and increased paracrine stimulation with thromboxane A_2_, owing to higher platelet density in the forming platelet aggregates.

Since bivalirudin retains binding to exosite I on thrombin after proteolytic degradation, we hypothesized that the delayed phase of platelet activation was caused by PAR4 activation. This notion was supported by the observations that (i) the PAR4 inhibitor BMS strongly inhibited the delayed phase of platelet activation observed with bivalirudin treatment; and (ii) that no additional inhibitory effect was observed when bivalirudin was combined with the PAR1 inhibitor VPX (Figures 2A–F). These findings show that the delayed phase of platelet activation observed with bivalirudin is primarily derived from PAR4. PAR1 inhibition with vorapaxar produced a less pronounced inhibitory effect on thrombin-triggered platelet aggregation in the presence of bivalirudin during the first 14 min of thrombin exposure, but at 1 μg/mL bivalirudin, this effect could only be observed in conjunction with stimulation from PAR4, as essentially no platelet activation response was observed with a combination of bivalirudin and BMS.

When the bivalirudin concentration was lowered to 0.5 μg/mL, mimicking drug levels observed ~2 h after termination of drug infusion at the recommended dosing during PCI (17), we observed a biphasic calcium response, with platelet calcium mobilization mainly occurring at 0–3 and 5–10 min. Moreover, rapid PAR1-mediated platelet activation occurred even without concurrent PAR4 signaling, as demonstrated by the disappearance of the initial calcium spike upon co-treatment with vorapaxar (Supplementary Figure 3). These findings show that thrombin stimulation in the presence of bivalirudin results in biphasic platelet activation with sequential signaling from PAR1 and PAR4. At therapeutic bivalirudin concentrations, the first PAR1-derived activation phase is sufficiently attenuated to prevent activation of GpIIb/IIIa and granule release. In contrast, the second PAR4-derived activation phase results in sufficient calcium mobilization to elicit a robust platelet response with significant P-selectin expression and platelet aggregation.

PAR4 stimulation has been shown to induce platelet activation responses characterized by high degrees of procoagulant membrane exposure (11, 26). This is of particular interest since increased procoagulant platelet formation in the vicinity of a vessel injury or a ruptured atherosclerotic plaque is likely to accelerate thrombin generation, increasing the risk for thrombotic complications. Considering our finding that bivalirudin treatment is associated with a delayed phase of PAR4-mediated platelet activation by thrombin, we used confocal fluorescence microscopy to investigate whether bivalirudin treatment is associated with decreased inhibition of procoagulant activity in thrombin-stimulated platelets adhering to fibrinogen. As shown in Figures 2G–I, our results demonstrate that although bivalirudin treatment inhibits the formation of Annexin V-binding procoagulant platelets directly after thrombin exposure, significant increase in procoagulant activity can be observed after prolonged incubation. This delayed procoagulant activity occurred in the same time interval as the late onset PAR4-derived sustained intracellular calcium elevation.

### Synergistic Thrombin Inhibition With a Combination of Unfractionated Heparin and Bivalirudin

A large proportion of patients that receive bivalirudin during PCI have already been treated with heparin, either peri-procedurally or as part of the pre-hospital management of myocardial infarction (2). Considering that the plasma half-life of heparin is 1–2 h, these patients will retain low but non-negligible residual plasma concentrations of heparin during the course of the intervention. Given our results that treatment with heparin was associated with profound and sustained inhibition of thrombin-induced platelet activation via PAR4, we hypothesized that this residual heparin could have a synergistic inhibitory effect on the PAR4-derived delayed phase of platelet activation observed with bivalirudin. As shown in Figures 2D–F,J–L, addition of a small dose of heparin (0.2 U/mL) abolished the delayed platelet activation observed with bivalirudin, demonstrating that the complementary inhibitory activities of these drugs with respect to PAR1 and PAR4 results in profound inhibition of thrombin-induced platelet activation when used in combination.

## Discussion

**Figure 3:**
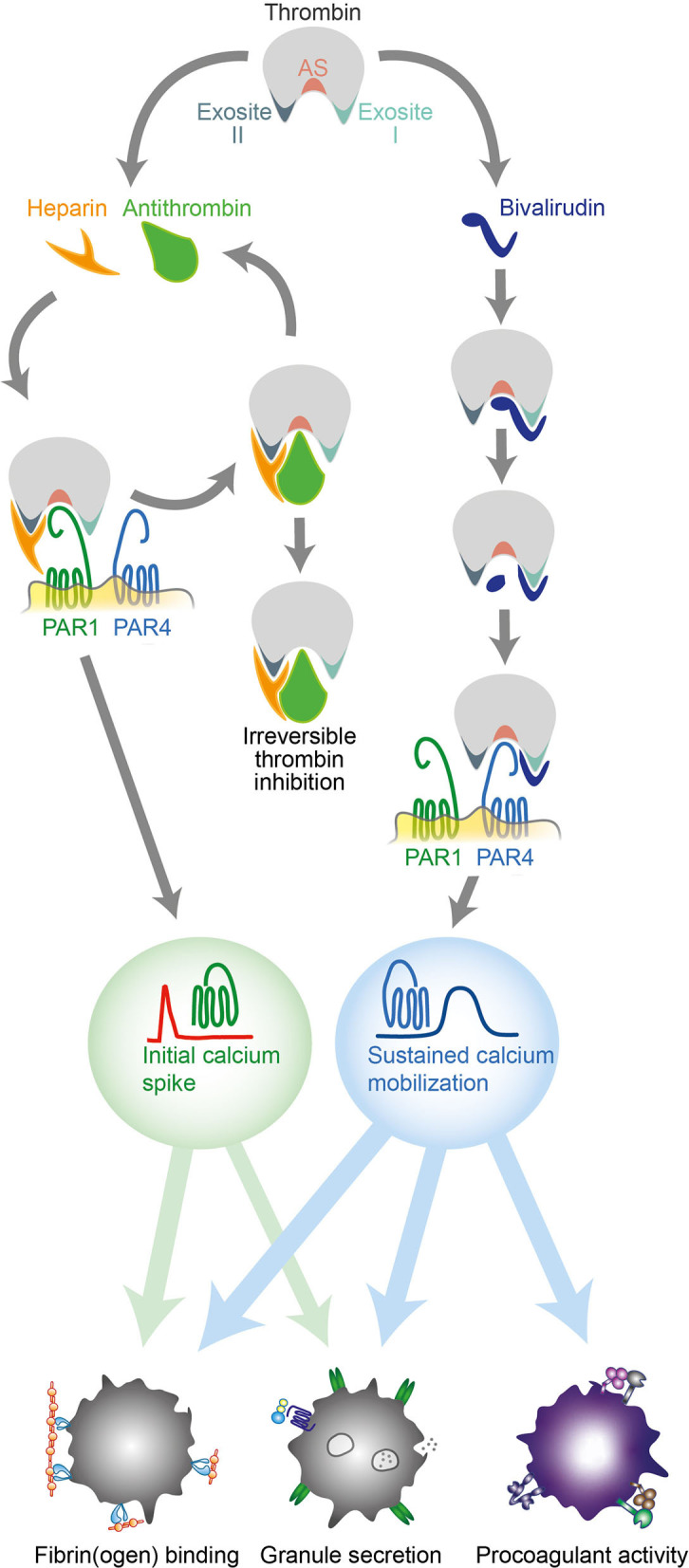
Different effects of heparin and bivalirudin on thrombin-induced platelet activation via PAR1 and PAR4. Schematic drawing illustrating the early and late inhibitory effects of heparin/antithrombin and bivalirudin on thrombin-induced platelet activation. AT, antithrombin; PAR, protease activated receptor; Thr, thrombin.

The importance of thrombin-induced platelet activation via PAR1 and PAR4 in the pathogenesis of thrombosis has been demonstrated both experimentally (27, 28) and clinically (29–31). However, despite contributions on the subject (17), our knowledge of how clinically important thrombin inhibitors modulate the platelet response to thrombin by interfering with PAR receptor activation remains rudimentary. In this study, we identify marked differences in the inhibitory effects of heparin and bivalirudin on thrombin-induced platelet activation via PAR1 and PAR4. Further, we show that these differences generate distinct prothrombotic responses in platelets exposed to high concentrations of thrombin (Figure 3). While thrombin inhibition with heparin/antithrombin was found to efficiently block PAR4 activation, an initial phase of transient PAR1-derived signaling resulted in a moderate degree of residual platelet aggregation and α-granule secretion. In contrast, bivalirudin treatment was found to result in a biphasic platelet response to thrombin stimulation, wherein an initial phase of profound inhibition of platelet activation was superseded by a delayed PAR4-derived activation response with prolonged calcium mobilization, resulting in significant α-granule release, GPIIb/IIIa activation, platelet aggregation, and procoagulant platelet activity. Although the exact molecular mechanisms responsible for these differences are not identified herein, mechanistic insights can be inferred from previous studies in the field. The previously known interaction between heparin and thrombin's exosite II is likely to play an important role for the preferential inhibition of thrombin-induced PAR4 activation by heparin/antithrombin. Indeed, it has been shown that heparin and other molecules interacting with exosite II inhibit platelet aggregation induced by γ-thrombin (10), a proteolytic degradation product of thrombin that lacks exosite I and solely retains the capacity to cleave PAR4. Moreover, a low molecular weight synthetic heparin analog specifically designed to target exosite II has been found to inhibit thrombin-induced PAR4 activation (16). For bivalirudin, the biphasic PAR signaling response observed herein can be linked to thrombin's gradual proteolytic cleavage of bivalirudin, as this process leaves both exosite II and the active site free to interact with PAR4 while preserving bivalirudin's blockage of exosite I, a domain that is particularly important for PAR1 activation (32, 33).

To enable an adequate assessment of the capacity of heparin and bivalirudin to inhibit PAR-mediated platelet activation in the setting of acute thrombosis, the thrombin concentrations selected for experimentation should ideally correspond to those encountered in the thrombus micro-environment. Although reliable empirical data on intra-thrombus thrombin levels *in vivo* are currently lacking, rigorously performed measurements using *in vitro* flow chamber models estimate that thrombin levels reach ~15 μmol/L in the thrombus core (19). Out of this vast pool of enzymatically active thrombin, around 98.5% is captured by fibrin, leaving 20–200 nmol/L (~3–30 U/mL) soluble thrombin free to diffuse out of the thrombus and cause further thrombus expansion. This finding helps to give some informative context to a previous study on the effects of heparin and bivalirudin on PAR signaling, wherein no significant platelet activation could be detected in blood samples obtained from PCI patients treated with either heparin or bivalirudin after exposure to 0.2 U/ml thrombin (34). By using a significantly higher thrombin concentration in our experiments (4 U/ml or ~30 nmol/L), we believe that our data is likely to be more representative of the inhibitory effects of heparin and bivalirudin at the site of most therapeutic relevance.

Our observation that even a small dose of heparin was sufficient to completely quench the PAR4-derived delayed phase of platelet activation that occurs with bivalirudin provides a potential mechanistic explanation for the finding that bivalirudin was independently associated with a higher rate of stent thrombosis during PCI in clinical trials wherein pre-procedural use of heparin either constituted an exclusion criteria or occurred at low frequency (3, 35), but not in the recent VALIDATE study, wherein >90% of patients in the bivalirudin treatment arm also received a low dose of heparin (2). It also suggests that a small dose of heparin, added when the bivalirudin infusion is tapered after PCI, could prevent the increased incidence of periprocedural stent thrombosis observed in patients treated with bivalirudin. Additionally, the additive effect of heparin and bivalirudin observed herein suggests that a combination of these two common anticoagulants could be a feasible alternative in high-risk situations where single-drug treatment is insufficient to adequately prevent thrombosis. As our study shows that heparin provides a more potent inhibition of thrombin-induced PAR4 activation than bivalirudin, heparin could provide a more attractive therapeutic option in patients from ethnic groups with a high prevalence of genetic variants rendering carriers hyperreactive to PAR4 stimulation. Although the allele frequency of one of these variants varies widely across sub-Saharan African populations (36), recent studies have shown that they are overrepresented among Americans with self-identified ethnicity as “black” (37, 38), a group with an unproportionally high cardiovascular disease burden and worse survival after acute coronary syndrome events. Naturally, due to the limited scope of this study, these interpretations need to be validated in clinical trials.

Given the growing interest in PAR inhibitors as pharmacological targets for antithrombotic treatment (25, 26, 31, 39), an increasing number of patients receiving medications that interfere with these signaling pathways are likely to be treated with thrombin inhibitors in the future. Our study provides new insights into the potential compatibility of PAR inhibitors with heparin and bivalirudin that may prove valuable in future research efforts to tailor antithrombotic therapy to the risk profiles of individual patients.

## Data Availability Statement

The raw data supporting the conclusions of this article will be made available by the authors, without undue reservation.

## Ethics Statement

The studies involving human participants were reviewed and approved by The local Ethics Committee in Linköping, Sweden, ethical permission numbers 2012/382-31 and 2016/314-32. Written informed consent for participation was not required for this study in accordance with the national legislation and the institutional requirements.

## Author Contributions

NB conceived the study. ML, AM, and KT performed the experiments. NB, ML, TL, and AM designed the experiments, analyzed the data, and wrote the manuscript. All authors revised the manuscript and approved the final version.

## Funding

This work was supported by the Swedish Heart-Lung Foundation (2017-0440 and 2019-0370 to TL) and (2017-0318 to NB), the Swedish Research Council (2017-01177 to TL), Lions Research Fund to NB, and Region Östergötland to ML, NB, and TL.

## Conflict of Interest

The authors declare that the research was conducted in the absence of any commercial or financial relationships that could be construed as a potential conflict of interest.

## Publisher's Note

All claims expressed in this article are solely those of the authors and do not necessarily represent those of their affiliated organizations, or those of the publisher, the editors and the reviewers. Any product that may be evaluated in this article, or claim that may be made by its manufacturer, is not guaranteed or endorsed by the publisher.
