# Supplementary material for: Effects of Heparin and Bivalirudin on Thrombin-Induced Platelet Activation: Differential Modulation of PAR Signaling Drives Divergent Prothrombotic Responses
Source: Front Cardiovasc Med. 2021 Sep 29;8:717835. doi: 10.3389/fcvm.2021.717835 (PMC8511449; doi:10.3389/fcvm.2021.717835)
Supplement: Supplementary file 1 [file Data_Sheet_1.docx]

**Supplementary information**

**Effects of Heparin and Bivalirudin on Thrombin-Induced Platelet Activation: Differential Modulation of PAR Signaling Drives Divergent Prothrombotic Responses**

Mikael Lund, MD^1^, Ankit S. Macwan, PhD^1^, Kjersti Tunströmer, PhD^1^, Tomas L. Lindahl, MD, PhD^1, 2^, Niklas Boknäs, MD, PhD^1, 3^

1. Department of Biomedical and Clinical Sciences, Linköping University, Linköping, Sweden
2. Department of Clinical Chemistry and Department of Biomedical and Clinical Sciences, Linköping University, Linköping, Sweden
3. Department of Hematology and Department of Biomedical and Clinical Sciences, Linköping University, Linköping, Sweden

**Corresponding author**: Tomas L. Lindahl, Department of Biomedical and Clinical Sciences, Linköping University, 58183 Linköping, Sweden

e-mail: tomas.lindahl@liu.se, phone: +46 10 103 3227

**Supplementary figures**


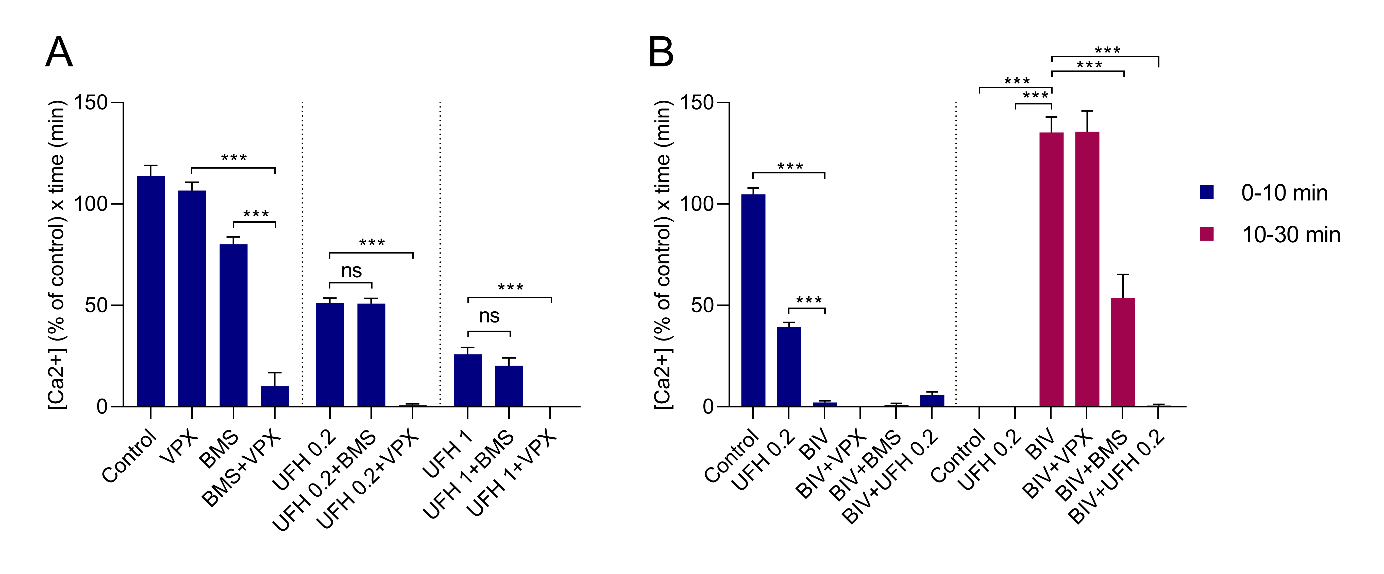


**Figure S1. Effects of heparin and bivalirudin on total platelet calcium mobilization tiggered by thrombin.** Total calcium mobilization (Area under the curve, AUC) was assessed by a spectrofluorometric method in suspensions of washed platelets supplemented with 1 U/mL antithrombin after addition of combinations of vorapaxar (5 µmol/L), BMS-986120 (100 nmol/L), heparin (0,2 and 1 U/mL), and bivalirudin (1 µg/mL). Platelets were activated with α-thrombin (4 U/mL). Data was normalized against the maximum signal obtained with the control (α-thrombin) at 0-10 min, and AUC was calculated for each combination. **A** shows the early effects on total calcium mobilication caused by PAR-inhibitors and heparin. **B** shows the early and late effects on total calcium mobilization caused by bivalirudin alone or in combination with PAR inhibitors and/or heparin. Results depict the mean +/- SEM. (In **A**: Control, VPX, n = 12; BMS, BMS+VPX, n = 8; UFH0.2, UFH1, n = 11; UFH0.2+BMS, UFH1+BMS, n = 7; UFH0.2+VPX, UFH1+VPX, n = 6. In **B**: Control, BIV, n = 21; UFH0.2, BIV+UFH0.2, n = 11; BIV+VPX, n = 18; BIV+BMS, n = 13). ***P< 0.001; not significant (ns). Abbreviations: VPX = vorapaxar, BMS = BMS-986120, UFH0.2 and UFH1 = unfractionated heparin (0.2 and 1 U/mL respectively), BIV = bivalirudin.


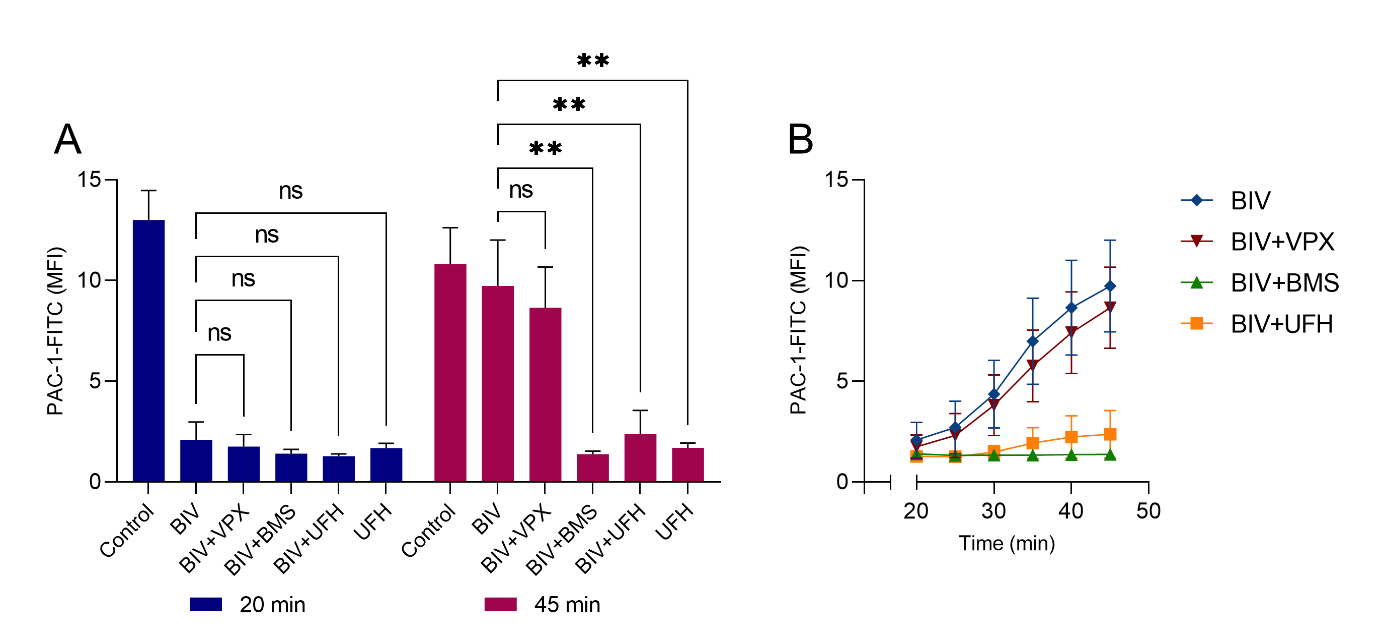


**Figure S2. Bivalirudin is associated with late-onset GPIIb/IIIa activation due to stimulatory signalling from PAR4.** Activation of GPIIb/IIIa was determined with flow cytometry by measuring binding of mAb PAC-1 20-45 minutes after exposure to 4 U/mL α-thrombin. **A** displays the effects of bivalirudin, PAR-inhibitors and heparin 20 and 45 min after activation with thrombin. **B** shows the temporal changes 20-45 minutes after activation with thrombin. Results depict the mean +/- SEM. (Control, BIV, BIV+VPX, BIV+BMS, BIV+UFH, n =12; UFH, n =10). **P< .01; not significant (ns). Abbreviations: VPX = vorapaxar (5 µmol/L), BMS = BMS-986120 (100 nmol/L), UFH = unfractionated heparin (0.2 U/mL), BIV = bivalirudin (1 µg/mL).


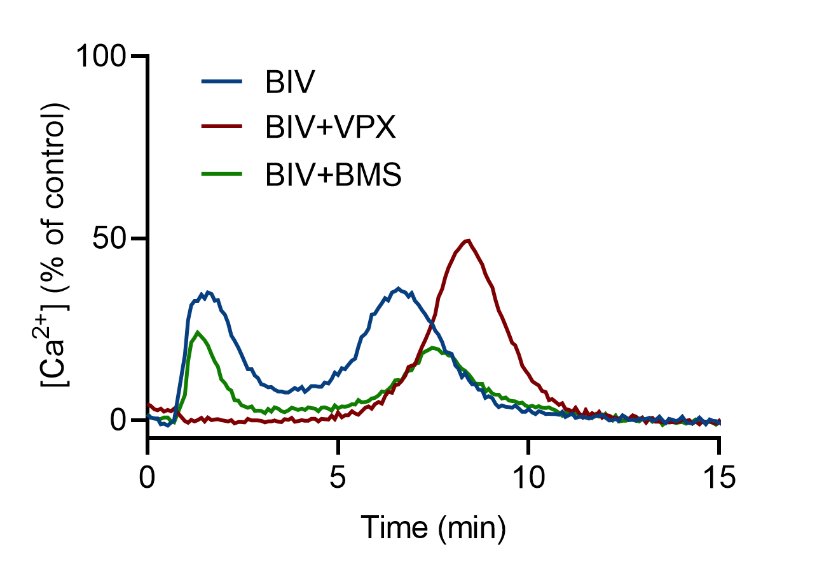


**Figure S3. Biphasic platelet calcium mobilization observed with a subtherapeutic concentration of bivalirudin.** As the bivalirudin concentration was lowered to 0.5 µg/mL, the biphasic kinetics of calcium mobilization became evident. The initial spike in calcium mobilization could be inhibited with a combination of bivalirudin and the PAR1 antagonist vorapaxar. Platelets were activated with 4 U/mL α-thrombin. Representative data is shown. Abbreviations: VPX = vorapaxar (5 µmol/L), BMS = BMS-986120 (100 nmol/L), BIV = bivalirudin (0.5 µg/mL).
